# Bibliometric analysis of publication trends and research hotspots in vagus nerve stimulation: A 20-year panorama

**DOI:** 10.3389/fneur.2022.1045763

**Published:** 2022-12-21

**Authors:** Rongrong Li, Hantong Hu, Ning Luo, Jianqiao Fang

**Affiliations:** ^1^Department of Acupuncture and Moxibustion, The Third Affiliated Hospital of Zhejiang Chinese Medical University, Hangzhou, Zhejiang, China; ^2^The Third Clinical Medical College, Zhejiang Chinese Medical University, Hangzhou, Zhejiang, China

**Keywords:** vagus nerve stimulation, neuromodulation, neurophysiology, bibliometrics, visualization

## Abstract

**Background:**

As a promising neuromodulation technique, vagus nerve stimulation (VNS) has been utilized to treat diverse diseases and the number of VNS studies has grown prosperously. Nonetheless, publication trends and research hotspots in this field remain unknown. This study aimed to perform a bibliometric analysis to systematically identify publication trends and research hotspots in VNS research within a 20-year panorama.

**Methods:**

The Web of Science Core Collection (WoSCC) database was retrieved to screen eligible VNS-related publications from 2002 to 2021. The online analytic tool of the WoSCC database was used to analyze various bibliometric parameters, such as the number of annual publications, the output of countries/regions, journals, total citations, citations per publication, and the Hirsch index. Bibliometrics (http://bibliometric.com/) and CiteSpace (version 5.6.R3) were used to identify research trends and hotspots.

**Results:**

A total of 7,283 publications were included for analysis. The annual number of publications increased stably but it increased significantly in recent years. The top five prolific countries were the United States, China, Germany, England, and France. The top five productive institutions were the *University of California (Los Angeles), Harvard Medical School, Harvard University, University College London*, and the *University of Texas at Dallas*. Notably, there was a geographical imbalance in countries and institutions. In addition, *Epilepsy & Behavior, Epilepsia*, and *Plos One* were the top three journals with the largest number of VNS publications. Michael P Kilgard was the most prolific author. Moreover, evolving research hotspots mainly included the effectiveness and mechanism of VNS on epilepsy, the role of VNS as an anti-inflammatory regulator, the application of VNS for psychiatric disorders, and the neuromodulation effect of VNS in headache management.

**Conclusion:**

This study has revealed the overall publication trends and evolving research trends at a global level over a 20-year panorama. The potential collaborators, institutions, hotspots, and future research trends are also identified in this field, which will help guide new research directions of VNS.

## Introduction

Vagus nerve stimulation (VNS) is a promising neuromodulation technique, administered *via* a minimally invasive or non-invasive approach. Invasive VNS can be implemented with surgically implanted electrodes, while non-invasive VNS (i.e., nVNS) can be performed by the transcutaneous auricular vagus nerve stimulation (taVNS) to stimulate the auricular branch of the vagus nerve with electrodes placed over specified sites (1). The rationale for selecting the vagus nerve as an attractive target for stimulation to treat a variety of diseases (especially, neurological and psychiatric disorders) is mainly because the wandering path of the vagus nerve intensively communicates with multiple brain structures and visceral organs. Dating back to 1997, VNS has been proven by the United States Food and Drug Administration (FDA) for treating epilepsy (2). Notably, as a non-pharmaceutical, effective, user-friendly, and well-tolerated therapy, taVNS has been widely used for various diseases with numerous benefits. In addition, taVNS has been investigated as a promising approach for treating a wide range of potential therapeutic indications over the past decades, including, but not limited to, headache (3), migraine (4), depression (5), insomnia (6), tinnitus (7), and post-stroke motor speech disorders (8).

Bibliometric analysis, as a well-established quantitative method for analyzing and quantifying literature information, has been adopted in diverse research fields to identify relevant bibliometric parameters (9), such as core scholars/institutions/countries and the cooperative association among them, co-occurrence analysis of keywords, the burst of keywords, and co-citation analysis (10). Such bibliometric parameters and information can significantly contribute to revealing the current status, topic hotspots, and research trends over time in a specific research field (9). Multiple research fields, such as pain medicine (11), anesthesiology (12), neurology (6), respiratory medicine (13), cancer medicine (14), and neuroimaging (15), have adopted this analysis approach to analyze the publication trend on a national or global level.

Although the number of VNS studies continues to grow rapidly in the research field of neuromodulation, to the best of our knowledge, an in-depth bibliometric analysis of VNS has not yet been conducted. Therefore, this study aimed to utilize a bibliometric analysis on VNS for the first time to systematically explore its publication trends and research hotspots over a 20-year panorama, which will further shed the light on future research in this field.

## Materials and methods

### Source of data and search strategy

Publications were retrieved from the Web of Science Core Collection (WoSCC) database with the following search strings: Topic = (Vagus Nerve Stimulation) OR (Auricular Vagus Nerve Stimulation) OR (Non-invasive Vagus Nerve Stimulation) OR (GammaCore) OR (Nemos), with the limited time frame set from 2002 to 2021. To reduce the deviations caused by daily updates of the database, publications retrieval and data downloads were all accomplished on 20^th^ July 2022. Only original articles and reviews were included, and other study types, such as meeting abstracts, editorial materials, letters, and corrections, were excluded. However, there were no geographic or language restrictions.

### Data collection and analysis

All data were retrieved by two authors (RRL and HTH) from the WoSCC. First, the retrieved data were analyzed using the WoSCC online analysis tool to yield various bibliometric parameters, mainly including the number of annual publications, the output of countries/regions, journals, total citations, citations per publication (CPP), and the Hirsch index (H-index). To the best of our knowledge, the H-index is widely used to quantify and standardize the scientific impact of researchers (16), and also accurately reflect the academic achievements of a country or an author; the influence of a study usually increases with the rise of the H-index (17). The impact factor (IF) and quartile of a journal category were obtained from the Journal Citation Reports (JCR) 2021. Any disagreements were resolved by judgment from a third senior reviewer (JQF).

Second, the website of bibliometrics (http://bibliometric.com/) and CiteSpace (version 5.6.R3) software were used to analyze and predict publication trends and hotspots in this field. Specifically, the website of bibliometrics was used to plot the collaboration analysis of countries/regions. CiteSpace (version 5.6.R3) is a tool designed for conducting a visual analytic study of scholarly literature in a specific research field, or a discipline, collectively known as a knowledge domain (18). All the downloaded data were exported into the CiteSpace (version 5.6.R3) to analyze diverse important bibliometric parameters, including co-citation analysis of the countries, journals, institutions, authors, and references, as well as the timeline view of co-cited references. In addition, the keywords with strong citation bursts and cluster maps of all items were captured.

The parameters of CiteSpace (version 5.6.R3) were set as follows: time-slicing was from 2002 to 2021, years per slice (1), all options in the term source were chosen, selected one node type at a time, and selection criteria were top 50 objects. Visualization knowledge figures consist of nodes and links. Each node in the map represented an element, including country, institution, co-cited author, co-cited reference, and keywords. The size of the node represented the frequency of appearance or citation, and the different colors of the nodes indicated the different years. The circles of the different colors from the inside to the outside of the node represented the years from 2002 to 2021. The nodes in the outermost area with a purple ring indicated high centrality, which was usually considered pivotal points or key points in a specific research field. In addition, lines between the nodes revealed cooperation, co-occurrence, or co-citation relationships.

Given that publicly available data were retrieved to perform the bibliometric analysis in this study, and no private information of individuals was involved, an ethical review by an institutional review board was not required.

## Results

### Annual number of publications and citations

**Figure 1:**
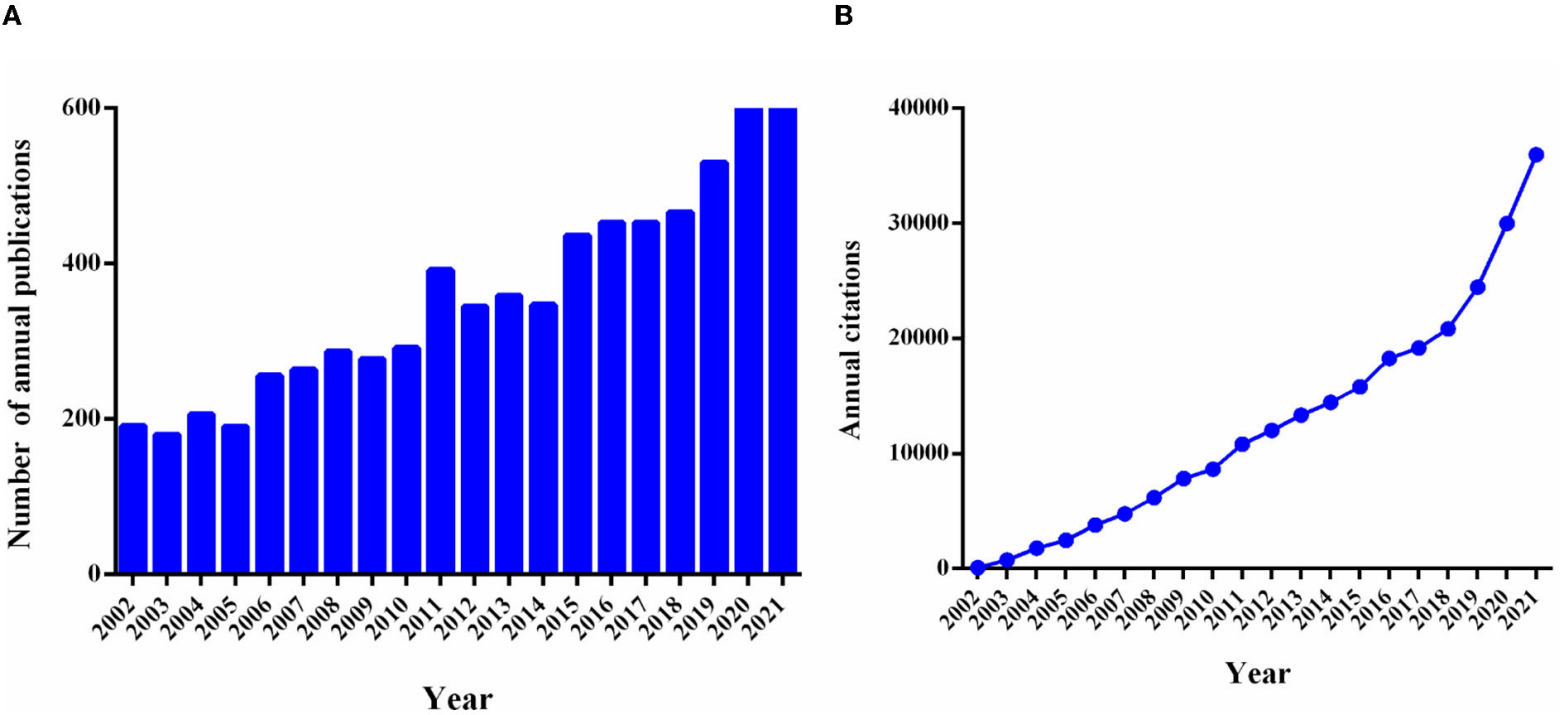
Trends in the number of annual publications and citations in VNS research from 2002 to 2021. **(A)** The trends of annual global publication outputs and the bar chart reflect the number of articles online per year. **(B)** The trends of annual global citations; exported the results from the GraphPad Prism (Version 6.01).

A total of 7,283 non-duplicate publications over the period from 2002 to 2021 were included in the bibliometric analysis, including 5,965 articles and 1,318 reviews. The growth of the annual publication output and the annual citations were shown in Figure 1. Compared with 2002 (*n* = 191), the growth rate of publication counts in this field was 100.05% in 2011 (*n* = 392). Notably, as shown in Figure 1, the annual number of articles and citations increased significantly in the past 10 years, reaching the highest point in 2021 (*n* = 697; citations = 35,972). In addition, the top 10 cited publications were shown in Table 1. The most frequently cited publication was a review published in *Nature* in 2002 entitled “*The inflammatory reflex*” with 2,242 citations (19). The annual citation rate (ACR), which was calculated as the ratio between the number of citations and the number of years since its publication, is 106.76 citations/year for this article. The second most frequently cited article (cited 2,200 times and 110.00 citations/year) was “*Nicotinic acetylcholine receptor alpha 7 subunit is an essential regulator of inflammation*” by HC Wang et al., published in *Nature* in 2003 (20).

**Table 1:** The top 10 most cited publications in VNS research.

**Rank**	**Title**	**First author**	**Journal**	**Country**	**IF (2021)**	**Category Quartile**	**Publication year**	**Citations** ^a^	
								**Total**	**ACR**
1	The inflammatory reflex	Tracey KJ.	Nature	England	69.50	Q1	2002	2,242	106.76
2	Nicotinic acetylcholine receptor alpha 7 subunit is an essential regulator of inflammation	Wang, Haichao	Nature	England	69.50	Q1	2003	2,200	110.00
3	Normal gut microbiota modulates brain development and behavior	Heijtza, RD	Proc Natl Acad Sci USA	USA	12.77	Q1	2011	1,806	150.50
4	Activation and Function of the MAPKs and Their Substrates, the MAPK-Activated Protein Kinases	Cargnello, M	Microbiol Mol Biol Rev	USA	13.04	Q1	2011	1,650	137.50
5	Brain structural and functional abnormalities in mood disorders: implications for neurocircuitry models of depression	Drevets, WC	Brain Structure & Function	Germany	3.74	Q3	2008	1,415	94.33
6	TrackMate: An open and extensible platform for single-particle tracking	Tinevez, JY	Methods	USA	4.64	Q2	2017	1,160	193.33
7	NF-kappa B and the link between inflammation and cancer	DiDonato, JA	Immunol Rev	Denmark	10.98	Q1	2012	1,051	95.55
8	Neurocircuitry of Mood Disorders	Price, JL	Neuropsychophar macology	England	8.29	Q1	2010	1,028	79.08
9	Physiology and immunology of the cholinergic antiinflammatory pathway	Tracey KJ	Journal of Clinical Investigation	USA	19.45	Q1	2007	944	62.93
10	Mutations of optineurin in amyotrophic lateral sclerosis	Maruyama, H	Nature	England	69.50	Q1	2010	822	68.50

a The citations times was according to WoS Core Collection; ACR, annual citation rate, citation/year.

### Distribution of countries/regions and institutions

**Figure 2:**
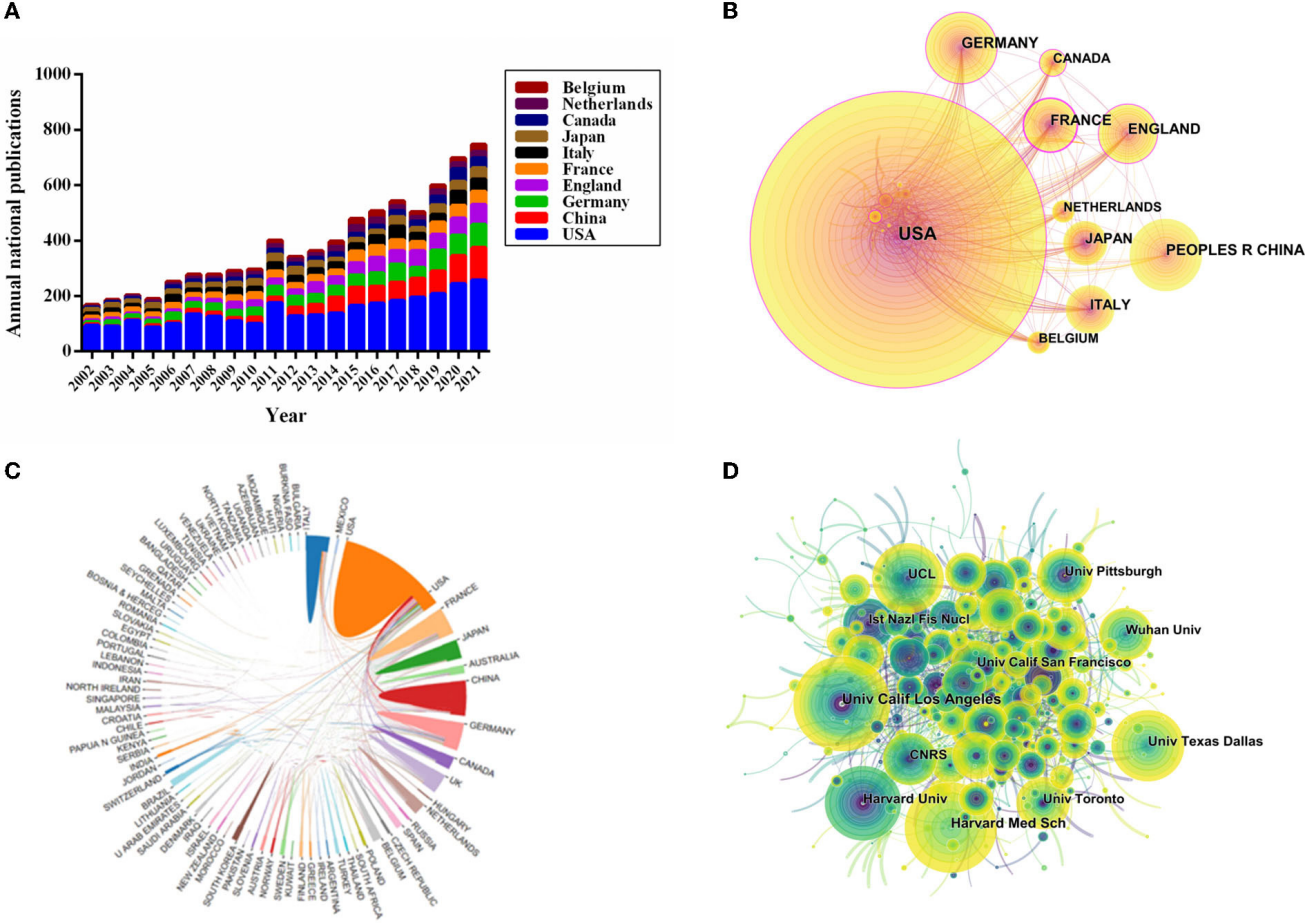
Visualization map of countries/regions and institutions related to VNS research from 2002 to 2021. **(A)** The annual national publication outputs and growth trends of the top 10 countries/regions *via* GraphPad Prism (Version 6.01). **(B)** CiteSpace collaboration network analysis of countries/regions. **(C)** The map of the international cooperation network in VNS research exported the results from the website of bibliometrics (http://bibliometric.com). **(D)** In collaboration network analysis of institutions *via* CiteSpace, the nodes in the map represent institutions, and the lines between the nodes represent collaboration relationships.

The distribution of all 7,283 publications covered 101 countries/regions. The top 10 countries of publications were demonstrated in Table 2. The United States ranked first in the number of publications (40.41% of 7,283 with 2,943 articles), followed by China (10.94%, with 797 articles), Germany (10.73%, with 782 articles), England (8.98%, with 654 articles), and France (7.99%, with 582 articles). Furthermore, the annual publications output of the 10 most productive countries/regions was identified. As shown in Figure 2A, the most prolific country for annual publications was the United States. It demonstrated that a leading position was maintained by the United States and it had a critical impact on the VNS research. Interestingly, since 2012, the fastest annual growth rate of publications has been in China, indicating that Chinese researchers had increased their enthusiasm in this research field in recent years and more relevant studies were carried out.

**Table 2:** The top 10 countries/regions of origin of papers in VNS research.

**Rank**	**Countries**	**Counts**	**% of 7,283**	**Citations WoS**	**Citations per paper**	**H-index**
1	USA	2,943	40.41	151,644	51.53	173
2	China	797	10.94	16,331	20.49	61
3	Germany	782	10.73	36,255	46.36	95
4	England	654	8.98	30,566	46.74	89
5	France	582	7.99	27,401	47.08	80
6	Italy	532	7.30	20,733	38.97	70
7	Japan	495	6.80	19,763	39.93	73
8	Canada	351	4.82	15,663	44.62	59
9	Netherlands	298	4.09	16,379	54.96	65
10	Belgium	283	3.89	12,203	43.12	58

International cooperation network analysis among different countries/regions in the VNS research was shown in Figures 2B, C. Further analysis revealed that the closest international collaborative relationship with the United States was with China, followed by Germany, the United Kingdom, France, Canada, and Japan.

Among the top 10 countries/regions of citations, the United States had the highest number of citations (151,644) and the highest value of the H-index (173). China ranked second in publications output (797), nonetheless, the citations (16,331), CCP (20.49), and H-index (61) were still at the lower aspects (Table 2).

The distribution of institutions map consists of 612 nodes and 1,258 links (Figure 2D), and a total of 612 institutions were dedicated to the research of VNS. Based on Figure 2D, close collaboration among institutions was still inadequate. The leading top five institutions of publications were the *University of California, Los Angeles* (1.52% with 111 articles), *Harvard Medical School* (1.43% with 104 articles), *Harvard University* (1.24% with 90 articles), *University College London* (1.19% with 87 articles), and the *University of Texas at Dallas* (1.15% with 84 articles), respectively. The 10 most productive institutions were shown in Table 3. Interestingly, “*University of California, Los Angeles*” was not only the most prolific institution but also the highest centrality institution.

**Table 3:** The top 10 institutions contributed to publications in VNS research.

**Rank**	**Institution**	**Count**	**Rank**	**Institution**	**Centrality**
1	Univ Calif Los Angeles	111	1	Univ Calif Los Angeles	0.11
2	Harvard Med Sch	104	2	NYU	0.08
3	Harvard Univ	90	3	Karolinska Inst	0.08
4	UCL	87	4	UCL	0.07
5	Univ Texas Dallas	84	5	Univ Toronto	0.07
6	Univ Pittsburgh	67	6	Stanford Univ	0.07
7	Univ Toronto	67	6	Massachusetts Gen Hosp	0.07
8	CNRS	64	8	CNRS	0.06
8	Ist Nazl Fis Nucl	63	9	Univ Cologne	0.06
10	Wuhan Univ	62	9	Univ Pavia	0.06

### Distribution of journals

The retrieved publications in this study were published in 1,754 journals and the top 10 productive journals were shown in Table 4. *Epilepsy & Behavior* was the most productive journal for publishing VNS research (with 124 publications), which had an IF of 3.337 in 2021, followed by *Epilepsia* (112), *Plos One* (111), *Journal of Biological Chemistry* (90), and *Seizure-European Journal of Epilepsy* (87), respectively. Four journals were related to “epilepsy” among the top 10 prolific journals. Moreover, *Epilepsia* had the higher total citations (5,790), and the article published by *Heck CN* in the journal of *Epilepsia* had the most citations (356) (21), followed by Laxer KD published in *Epilepsy & Behavior* with 334 citations (22). *Journal of Biological Chemistry* listed the highest H-index (45), and the journal with the highest IF was *Brain Stimulation* (IF = 9.184) (Table 4).

**Table 4:** The top 10 scholarly journals published articles in VNS research.

**Rank**	**Journal**	**Count**	**IF2021**	**Total citations**	**CPP**	**Country**	**H-index**
1	Epilepsy & Behavior	124	3.337	3,911	31.54	USA	36
2	Epilepsia	112	6.740	5,790	51.70	USA	44
3	Plos One	111	3.752	3,002	27.05	USA	31
4	Journal of Biological Chemistry	90	5.486	5,296	58.84	USA	45
5	Seizure-European Journal of Epilepsy	87	3.414	2,391	27.48	England	28
6	Scientific Reports	82	4.996	1,372	16.73	England	23
7	Brain Stimulation	78	9.184	3,217	41.24	USA	33
8	Nuclear Instruments and Methods in Physics Research Section A: Accelerators, Spectrometers, Detectors and Associated Equipment	76	1.335	821	10.80	Netherlands	15
9	Autonomic Neuroscience-Basic & Clinical	71	2.355	1,475	20.77	Netherlands	21
10	Epilepsy Research	65	2.991	1,603	24.66	Netherlands	21

### Distribution of authors and cited authors

**Figure 3:**
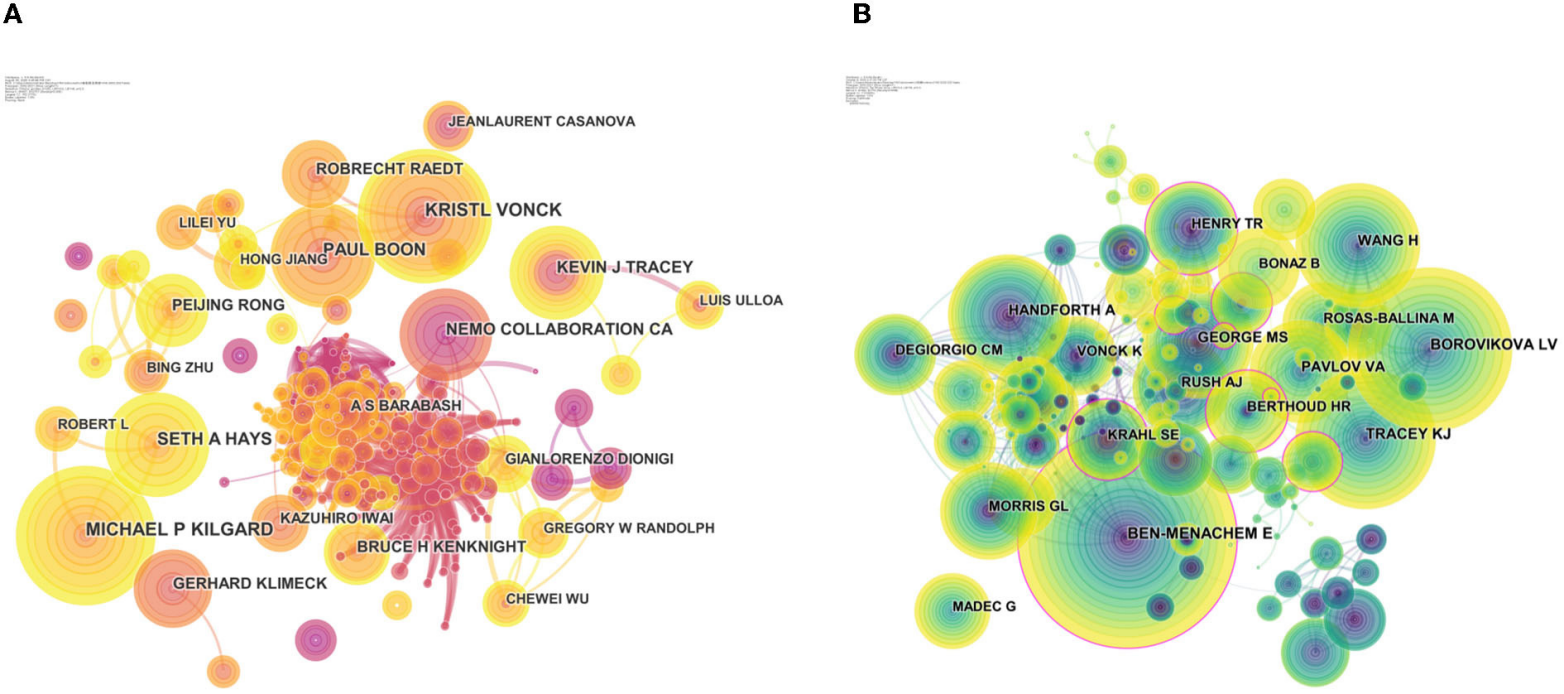
Visualization map of authors/cited authors. **(A)** CiteSpace visualization map of authors. **(B)** CiteSpace visualization map of cited authors. The nodes represent the authors and the lines between the nodes represent the collaboration relationships. The larger the node area, the larger the number of publications.

The authors of the 7,283 publications were analyzed, and this resulted in 947 nodes (Figure 3A). In terms of publications outputs, Michael P Kilgard was the most prolific author with 46 publications, followed by Kristl Vonck (45 publications), Paul Boon (35 publications), Seth A Hays (35 publications), and Kevin J Tracey (31 publications), and the top 10 productive authors are shown in Table 5. The map of authors revealed the closeness of collaborations among the authors, which could provide information on influential research groups and potential collaborators (Figure 3A). Based on Figure 3B, the closest cooperation was established in the teams of Ben-Menachem E and Borovikova LV as the core co-cited authors, respectively. Interestingly, they also had the most publication outputs in this field. In terms of the most co-cited authors, Ben-Menachem E ranked first with 846 citations, followed by Borovikova LV (652 citations), Tracey KJ (570 citations), Wang H (496 citations), and Handforth A (483 citations) (Table 5).

**Table 5:** The top 10 authors and co-cited authors in VNS research.

**Rank**	**Author**	**Count**	**Rank**	**Co-cited author**	**Frequency**
1	Michael P Kilgard	46	1	Ben-menachem E	846
2	Kristl Vonck	45	2	Borovikova LV	652
3	Paul Boon	35	3	Tracey KJ	570
4	Seth A Hays	35	4	Wang H	496
5	Kevin J Tracey	31	5	Handforth A	483
6	Nemo Collaboration CA	30	6	George MS	448
7	Gerhard Klimeck	26	7	Pavlov VA	412
8	Peijing Rong	24	8	Morris GL	400
9	Robrecht Raedt	23	9	Rush AJ	387
10	Bruce H Kenknight	23	10	Henry TR	367

### Distribution of cited references

**Figure 4:**
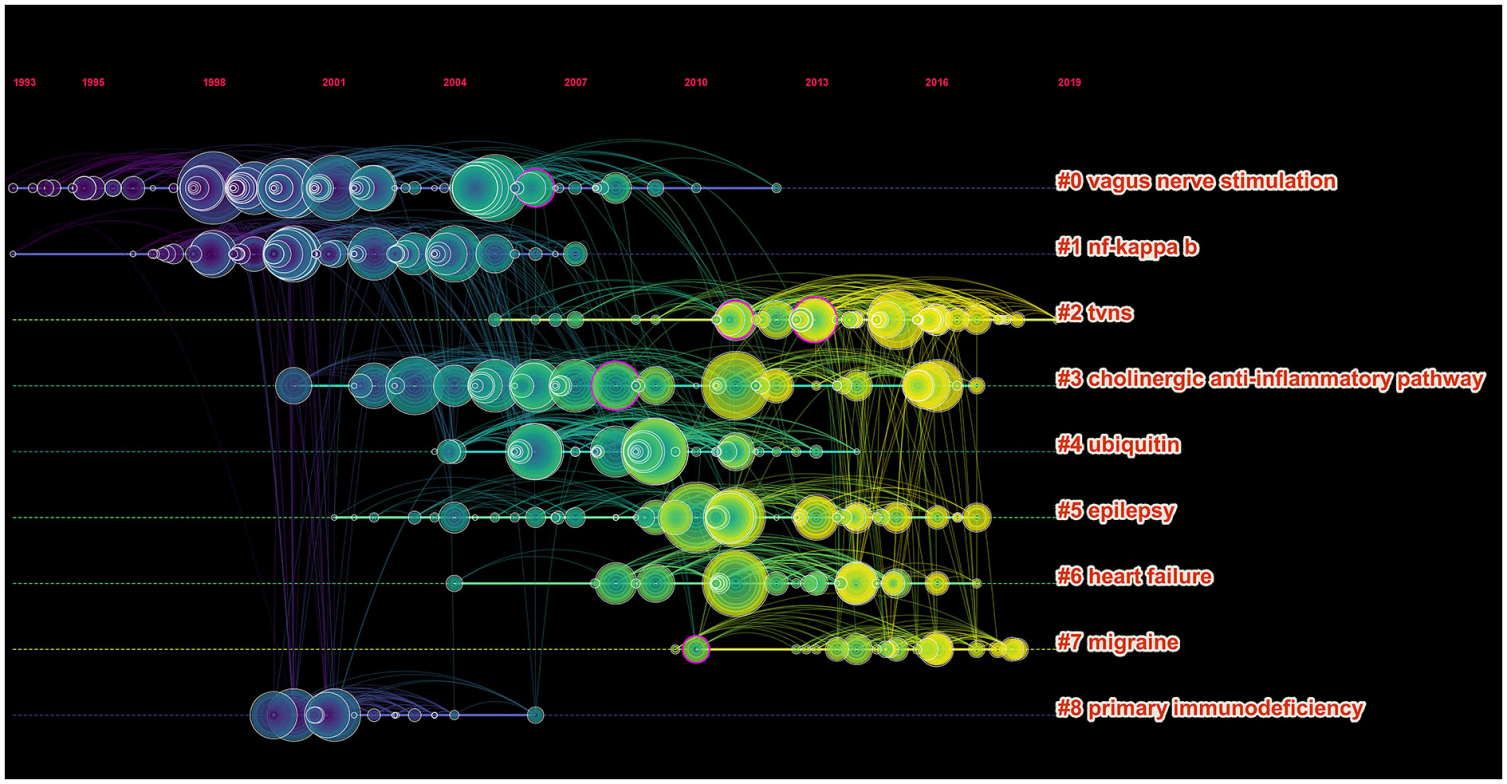
The timeline view clusters of co-cited references and their cluster labels in VNS research from 2002 to 2021, exported the results *via* CiteSpace, and the cluster with warmer colors and larger nodes contained more publications, indicating that this clustering issue was the hotspot in this field.

According to the five most frequently co-cited references (Table 6), the article entitled “*Acetylcholine-Synthesizing T Cells Relay Neural Signals in a Vagus Nerve Circuit*” by Rosas-Ballina and Mauricio was the most cited reference with 811 citations and it had the highest IF (63.71) as well (23). Additionally, the second most cited reference publication was “*Vagus nerve stimulation therapy for partial-onset seizures—A randomized active-control trial*” published in *Neurology* with 742 citations (24). Among the top five most frequently co-cited references, two other references were mainly related to anti-inflammatory (25) and improving the quality of life of patients with chronic heart failure (26). Furthermore, the timeline view map of co-cited references related to VNS research was executed by CiteSpace (version 5.6.R3) (Figure 4), and the log-likelihood rate (LLR) was used to find out the distribution of hotspots from references with nine clusters. The cluster with warmer colors indicated the latest research and larger nodes indicated more publications, indicating that this clustering issue was the hotspot in this field. Thus, cluster#2 (TVNS), cluster#3 (cholinergic anti-inflammatory pathway), cluster#5 (epilepsy), cluster#6 (heart failure), and cluster#7 (migraine) indicated the hotspots in VNS research during the past years. The structural characteristics of the cited references clusters were shown in Table 7, including the number of nodes in each cluster, silhouette, mean (year), and the name of clusters. It is generally believed that the cluster with a silhouette value >0.5 was reasonable, while a value >0.7 suggested that the cluster was convincing (27). The silhouette score was higher than 0.8 in all nine major clusters, which revealed the superior quality of clusters (Table 7). The highest silhouette was cluster #6 “heart failure” (0.971), with 26 publications. The biggest cluster was “vagus nerve stimulation”, including 87 nodes and its mean year of publication was 2000. Furthermore, the highest LLR was occupied by cluster #7 “migraine”, which contained 19 references with a rate of 76.93, followed by “heart failure” (55.38) and “cholinergic anti-inflammatory pathway” (37.89). In addition, the LLR of “epilepsy (24.7)” was ranked 7.

**Table 6:** The top five high-cited references in VNS research.

**Rank**	**References**	**Journal (JCR)**	**IF2021**	**First author**	**Publication time**	**Total citations**
1	Acetylcholine-Synthesizing T Cells Relay Neural Signals in a Vagus Nerve Circuit	Science	63.71	Rosas-Ballina, Mauricio	2011	811
2	Vagus nerve stimulation therapy for partial-onset seizures—A randomized active-control trial	Neurology	11.80	Handforth, A	1998	742
3	Vagus nerve stimulation inhibits cytokine production and attenuates disease severity in rheumatoid arthritis	Proc Natl Acad Sci USA	12.77	Koopman, Frieda A	2016	437
4	Chronic vagus nerve stimulation: a new and promising therapeutic approach for chronic heart failure	European Heart Journal	35.85	De Ferrari, Gaetano M	2011	322
5	Vagus nerve stimulation for epilepsy: a meta-analysis of efficacy and predictors of response A review	Journal of Neurosurgery	5.40	Englot, Dario J	2011	263

**Table 7:** The structural characteristics of the cited-references clusters.

**Cluster ID**	**Size**	**Silhouette**	**Mean (year)**	**Cluster name (LLR)**
#0	87	0.959	2000	Vagus nerve stimulation (22.2)
#1	52	0.806	2001	NF-Kappa B (29.14)
#2	47	0.803	2013	TVNS (29.28)
#3	47	0.969	2008	Cholinergic anti-inflammatory pathway (37.89)
#4	42	0.891	2008	Ubiquitin (34.91)
#5	40	0.924	2009	Epilepsy (24.7)
#6	26	0.971	2012	Heart failure (55.38)
#7	19	0.946	2014	Migraine (76.93)
#8	14	0.961	2002	Primary immunodeficiency (23.33)

The symbol “#” means “Cluster”.

### Distribution of keywords burst

**Figure 5:**
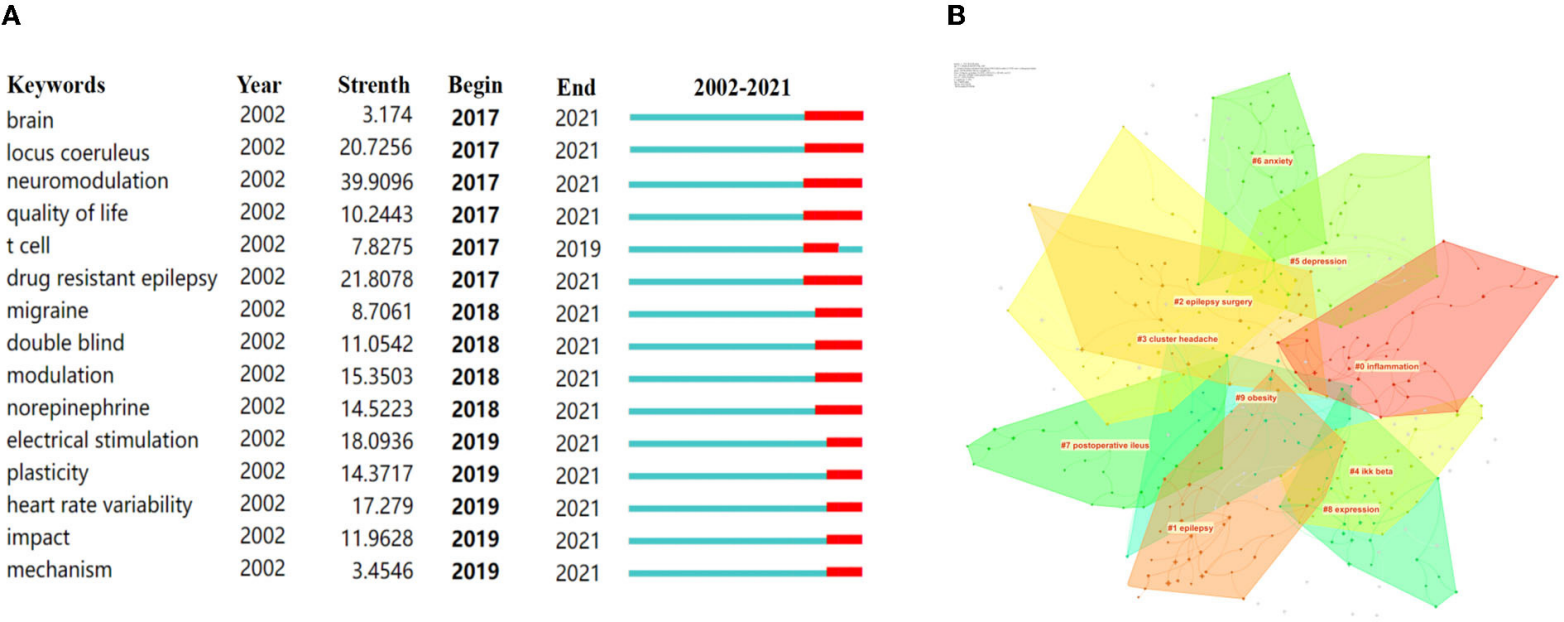
Keywords with the strongest citation burst and top 10 clusters over the past 5 years were exported *via* CiteSpace. **(A)** Keywords with the strongest citation bursts among the top 100 burst keywords related to VNS. **(B)** The 10 clusters of keywords related to VNS. The different colors mean different clusters.

All of the 831 keywords were generated by using CiteSpace (version 5.6.R3). The top 20 keywords in terms of frequency are presented in Table 8. Notably, “epilepsy” was the most frequent keyword (a frequency of 781) in VNS research during the past 20 years, except for the keywords of “vagus nerve stimulation” and “vagus nerve” (28–30). In addition, among the top 100 keywords with the strongest citation bursts, we mainly focused on those keywords that have burst from the past 5 years (Figure 5A) to identify hotspots and research frontiers that change over time (27). The top five burst strength keywords were defined, including “neuromodulation”, with the highest burst strength of 39.9096, followed by “drug-resistant epilepsy” (21.8078), “locus coeruleus” (20.7256), “electrical stimulation” (18.0936), and “heart rate variability” (17.279). In addition, clustering analysis was implemented to reveal the emerging trends in the last 5 years. According to Figure 5B, 10 clusters were identified, including “inflammation”, “epilepsy”, “cluster headache”, “depression”, “anxiety”, “postoperative ileus”, “obesity”, etc.

**Table 8:** Top 20 frequency of keywords in VNS research.

**Rank**	**Keywords**	**Frequency**	**Rank**	**Keywords**	**Frequency**
1	Vagus nerve stimulation	1971	11	Expression	379
2	Vagus nerve	908	12	Refractory epilepsy	377
3	Epilepsy	781	13	Children	349
4	Activation	737	14	Rat	323
5	Stimulation	556	15	Neuromodulation	313
6	Nemo	536	16	Deep brain stimulation	300
7	Electrical stimulation	474	17	Heart rate variability	282
8	Seizure	472	18	Efficacy	278
9	Inflammation	433	19	Mechanism	267
10	Therapy	422	20	Depression	257

## Discussion

Although some previous reviews have examined the application of VNS, all of them were limited to exploring its application to specific diseases (31–35). Hence, the key questions concerning publication trends, research hotspots, and other major bibliometric outcomes in the VNS field need to be urgently solved. Given the urgency of the situation, this study conducted a bibliometric analysis that mainly aimed to quantify and visualize publication trends and research hotspots in the VNS field within a 20-year panorama for the first time.

### General information

The annual number of publications and citations in the VNS field increased significantly. Notably, the United States was the main driving force with a high academic status in VNS research, which was confirmed by the highest number of annual publication outputs, as well as the highest citations and the H-index. However, the existence of geographical imbalance in this field should be noted. Only one developing country (i.e., the People's Republic of China) was featured in the top 10 prolific countries over the past 20 years. Therefore, there is an urgent need for developing countries to perform more close cooperation with developed countries to improve their academic status in this field.

In addition, the top three prolific institutions, including the “*University of California Los Angeles*,” “*Harvard Medical School*,” and “*Harvard University*,” pertained to the United States, given that the academic status of the United States in VNS research has been significantly enhanced through close cooperation between these institutions.

### References analysis

Among the analysis of cited references, cholinergic anti-inflammatory pathways, heart failure, and migraine were the hotspots in the field of VNS research over the past years. It was considered that VNS could yield anti-inflammatory effects by modulating the production of TNF, thus suggesting its potential value in the treatment of autoimmune and auto-inflammatory diseases by analyzing the most cited references. Additionally, other cited references also have shown that VNS could decrease fatigue and pain in patients with systemic lupus erythematosus (36) and rheumatoid arthritis (37), and provide benefits for inflammation-related gastrointestinal diseases (33). However, it is noteworthy that some research questions remain inconclusive, such as optimal parameters, duration, and stimulation regions of VNS. Given this scenario, randomized controlled trials with large sample sizes are mandatory to yield definitive answers to these questions.

### Evolution of research hotspots

The research hotspots are summarized below through an effective combination of top keywords, keywords burst, and cluster analysis. First, the effectiveness and mechanism of VNS for epilepsy are always the research hotspots in this field. As an optimized referral therapeutic option, VNS can reduce seizure frequency and severity in patients with refractory postencephalitic epilepsy (38), whereas the number of studies on optimized stimulation parameters remains insufficient (39). In addition, an updated guideline suggested that VNS may be considered for children with seizures (40). The mechanism of VNS on epilepsy possibly involves modulation of the locus coeruleus, thalamus, and limbic circuit through noradrenergic and serotonergic projections (41, 42). Nonetheless, the specific changes in these cortical circuits remain unknown. Therefore, future research perspectives can focus on (1) revealing the precise mechanism of VNS for epilepsy and (2) improving the efficacy and reducing the side effects of VNS for epilepsy.

Second, the role of VNS as an anti-inflammatory regulator is another emerging research hotspot. Increasing evidence demonstrated that VNS may attenuate inflammatory responses by activating the cholinergic anti-inflammatory pathway (CAP) (19, 43, 44). These findings support the critical role of VNS in the treatment of peripheral cholinergic release and its putative role in the inhibition of inflammation. Furthermore, accumulating evidence has revealed that VNS may be beneficial for multiple peripheral and central inflammatory disorders, such as rheumatic arthritis and irritable bowel syndrome (42, 45). However, further studies are urgently needed to fully elucidate the mechanism underlying the potential role of VNS in the treatment of these inflammatory diseases.

Third, recent research trends are evolving toward the application of VNS for psychiatric disorders, such as anxiety and depression. Nahas et al. revealed that VNS may have a long-term beneficial effect in patients with chronic or recurrent major depressive disorders (46). Additionally, the transmission of neurotransmitters in the medial and prefrontal cortex could be altered by VNS (including serotonin and norepinephrine), which exerts its anticonvulsive and antidepressant effects (47).

Fourth, the neuromodulation effect of VNS in headache management is another emerging research hotspot. As a major modality of neuromodulation techniques, VNS has been established as a safe and effective treatment option for migraine and cluster headaches (CH). It was identified that transcutaneous stimulation of the auricular branch of the vagal nerve (ta-VNS) at 1 Hz was effective for chronic migraine prevention over 3 months (48). In addition, the number of weekly attacks of cluster headaches was decreased significantly by daily nVNS treatment (49). Current widely accepted mechanisms underlying the effectiveness of nVNS in migraine and CH are mainly related to neurotransmitter regulation, nociceptive modulation, effects on autonomic nervous system functions, and inhibition of cortical spreading depression (CSD) (3). Notably, VNS may have therapeutic effects on migraine by inducing the release of norepinephrine and serotonin from the locus coeruleus and dorsal raphe nucleus, respectively, both of which are closely related to the pathogenesis of migraine (50). Given the accumulating evidence in recent years, VNS should be regarded as an important choice for headache management.

Finally, the relationship between VAS and acupuncture deserves attention. Interestingly, acupuncture, especially electroacupuncture (EA), has a certain comparability with VNS. As an important form of acupuncture, EA mainly exerts synergistic effects by needling acupoints and electrical stimulation to strengthen the therapeutic effects. Therefore, EA has similar neuromodulation effects as VNS and can be applied as a neuromodulation therapy. In addition, many classical acupoints are located in the suboccipital and lateral cervical regions, where the superficial branches of the vagus nerve are located (51). VNS has been introduced in the last two decades as a new treatment for refractory epilepsy and depression, while traditional Chinese medicine treats the same disorders by pricking the ear, mastoid, and suboccipital regions in anatomical proximity to the vagus nerve (51). Numerous studies indicated that acupuncture can modulate the activity of the parasympathetic nervous system directly or indirectly by stimulating the vagus nerve (52–54). Therefore, the fields of acupuncture research can refer to VNS studies for a more in-depth analysis of stimulation parameters and anatomical basis, thereby improving the scientific connotation of acupuncture research in future.

### Limitations

First, the data were extracted only from the WoSCC, and articles published in other sources (e.g., PubMed and Scopus) may be missed. However, WoSCC is a comprehensive, multidisciplinary, core journal citation index database, which mainly includes the references of the core journals, and it contains six sub-databases, such as Science Citation Index-Expanded. Therefore, the findings of this study are robust. Second, to analyze the full annual data, the time frame of included publications was limited to 2002 and 2021, and publications published in 2022 were not considered. Therefore, hotspots in the VNS field in 2022 may be missed. Nonetheless, the general publication trends and evolving research hotspots in VNS were identified by analyzing publications within a 20-year panorama.

## Conclusion

This bibliometric study has revealed the overall publication trends and evolving research trends at a global level over a 20-year panorama. The potential collaborators, institutions, hotspots, and future research trends are also identified in this field, which will help guide new research directions of VNS. All the findings will also guide clinicians, researchers, industry collaborators, policymakers, and other stakeholders.

## Data availability statement

The original contributions presented in the study are included in the article/Supplementary material, further inquiries can be directed to the corresponding author.

## Author contributions

All authors made substantial contributions to the conception and design, acquisition of data, or analysis and interpretation of data, took part in drafting the article or revising it critically for important intellectual content, gave final approval of the version to be published, and agree to be accountable for all aspects of the work.
